# Temperament and Character in the Child and Adolescent Twin Study in Sweden (CATSS): Comparison to the General Population, and Genetic Structure Analysis

**DOI:** 10.1371/journal.pone.0070475

**Published:** 2013-08-05

**Authors:** Danilo Garcia, Sebastian Lundström, Sven Brändström, Maria Råstam, C. Robert Cloninger, Nóra Kerekes, Thomas Nilsson, Henrik Anckarsäter

**Affiliations:** 1 Centre for Ethics, Law and Mental Health, University of Gothenburg, Gothenburg, Sweden; 2 Institute of Neuroscience and Physiology, The Sahlgrenska Academy, University of Gothenburg, Gothenburg, Sweden; 3 Swedish Prison and Probation Service, Research & Devolopment unit, Gothenburg, Sweden; 4 Gillberg Neuropsychiatry Centre, Institute of Neuroscience and Physiology, The Sahlgrenska Academy, University of Gothenburg, Gothenburg, Sweden; 5 Department of Clinical Sciences, Lund University, Lund, Sweden; 6 Departments of Psychiatry & Genetics, Washington University School of Medicine in St. Louis, St. Louis, Missouri, United States of America; 7 Department of Clinical Sciences, Lund University, Malmö, Sweden; University of Hong Kong, Hong Kong

## Abstract

**Background:**

The Child and Adolescent Twin Study in Sweden (CATSS) is an on-going, large population-based longitudinal twin study. We aimed (1) to investigate the reliability of two different versions (125-items and 238-items) of Cloninger's Temperament and Character Inventory (TCI) used in the CATSS and the validity of extracting the short version from the long version, (2) to compare these personality dimensions between twins and adolescents from the general population, and (3) to investigate the genetic structure of Cloninger's model.

**Method:**

Reliability and correlation analyses were conducted for both TCI versions, 2,714 CATSS-twins were compared to 631 adolescents from the general population, and the genetic structure was investigated through univariate genetic analyses, using a model-fitting approach with structural equation-modeling techniques based on same-sex twin pairs from the CATSS (423 monozygotic and 408 dizygotic pairs).

**Results:**

The TCI scores from the short and long versions showed comparable reliability coefficients and were strongly correlated. Twins scored about half a standard deviation higher in the character scales. Three of the four temperament dimensions (Novelty Seeking, Harm Avoidance, and Persistence) had strong genetic and non-shared environmental effects, while Reward Dependence and the three character dimensions had moderate genetic effects, and both shared and non-shared environmental effects.

**Conclusions:**

Twins showed higher scores in character dimensions compared to adolescents from the general population. At least among adolescents there is a shared environmental influence for all of the character dimensions, but only for one of the temperament dimensions (i.e., Reward Dependence). This specific finding regarding the existence of shared environmental factors behind the character dimensions in adolescence, together with earlier findings showing a small shared environmental effects on character among young adults and no shared environmental effects on character among adults, suggest that there is a shift in type of environmental influence from adolescence to adulthood regarding character.

## Introduction

Cloninger's psychobiological model of personality [1] consists of four temperament and three character dimensions. The temperament dimensions are defined in terms of individual differences in behavioral learning mechanisms: the behavioral activation system which reflects the tendency toward exploratory action and intense excitement in response to novel stimuli (Novelty Seeking); the behavioral inhibition system which reflects the tendency to respond intensely to aversive stimuli and to avoid punishment and novel stimuli (Harm Avoidance); the behavioral maintenance system which reflects the tendency to respond strongly to reward and to learn to maintain rewarded behavior (Reward Dependence); and the propensity to persevere in behaviors despite frustration and fatigue (Persistence). Character involves both neurobiological and sociocultural mechanisms of semantic and self-aware learning (i.e., self-concepts about goals and values or what people make of themselves intentionally). The three character dimensions are: Self-directedness, which indicates how responsible, purposeful, and resourceful an individual is in working to achieve her goals and values (i.e., the ability to identify the self as autonomous); Cooperativeness, which indicates how well adapted the individual is in getting along with others fairly and flexibly, with kindness (i.e., the ability to identify the self as an integral part of society); and Self-transcendence, which indicates transpersonal identification or conscience (i.e., the ability to identify the self as part of the whole universe and in union with all things) [2]. Factor analytic studies, based on self-reported temperament and character using the Temperament and Character Inventory (TCI), have supported Cloninger's seven-factor model of personality [3], [4].

Different versions of the TCI (e.g., 125-items, 238-items) have been validated against other measures of personality [5], temperament [6] and mental health [7], [8]. The TCI, for example, has been compared to 11 modern multi-scale personality inventories by independent investigators and consistently showed predictive validity as good or better as any other available test [9]. Moreover, the TCI has been translated into and validated in several languages, such as, Swedish [10], Dutch [11], Japanese [12], Turkish [13], and Spanish [14], [15]. These studies show sound psychometric properties comparable to what was found for the original version. The TCI has been widely used in the investigation of personality's neurobiological foundations, together with other research technologies, such as, molecular neuroimaging [16], structural neuroimaging [17], and genetics.

Twin studies suggest at least equally (if not more) important roles of genetic influence on the character dimensions as on the temperament dimensions. Ando and colleagues [18], for example, found among 296 Japanese twin pairs that Harm Avoidance and Persistence showed significant additive genetic contributions and no shared environmental effect, supporting the original theoretical assumption. Nevertheless, in their study, Novelty Seeking and Reward Dependence could be explained by both genetic and shared environmental factors. In contrast to Cloninger's assumptions, all three character dimensions could be explained exclusively by additive genetic and unique environmental contributions – unique environment is what makes twins growing up in the same family dissimilar rather than similar, while the common environment is what makes twins similar. Moreover, the genetic components of Self-directedness and Cooperativeness were derived from those of the temperament dimensions [18]. The character traits have indeed shown much the same levels of heritability as the temperament traits across several studies [19], [20]. Studies using adolescents, however, have not investigated the genetic structure of the character dimensions (e.g., [21], [22]).

Cloninger's psychobiological theory of personality provides a model that can be empirically verified by behavioral genetics methodology because it proposes that humans are an integrated hierarchy of biological, psychological, and social systems that adapt to changes [18]. Although the character dimensions are influenced by social norm-favoring [1], [23], no substantial support has been found to state that character is influenced by shared environmental effects in twin studies. Most twin studies, for example, point out that, at least in adolescence and adulthood, genes influence prosocial traits, such as Cooperativeness, as does the unique environment (for a review, see [24]). However, adolescence is a specific period in life that early on was suggested to be an important phase in the development of an individual's autonomous identity, social affiliations, and other goals and values that organize a person's lifestyle [25]. Indeed, adults and adolescents who report high levels of Self-directedness and Cooperativeness also report frequently experiencing positive emotions, high levels of satisfaction with life [26]–[33], and less psychosocial dysfunction and suffering [34]. In accordance with these findings, the occurrence of various forms of psychopathology, including affective and behavioral disorders, increases dramatically during adolescence [35]. More recently, neuroimaging research suggests that cognitive and behavioral changes occurring during adolescence might be understood from the perspective of increased “executive functioning” (e.g., attention, response inhibition, regulation of emotion, organization, and long-range planning; for a review, see [36]). This development of higher-order functioning relies on frontal lobe circuitry and emerges as a gradual maturing of the individual character.

### The present study

The present study was conducted using self-reported temperament and character measures from the Child and Adolescent Twin Study in Sweden (CATSS), which is an on-going, large population-based longitudinal twin study targeting all twins born in Sweden since July 1, 1992. By January 2010, the CATSS was comprised of around 17,000 twins and a response rate of roughly 80% (for a detailed description of the CATSS, see [37]). We focus on data from the 15-year-old twins' follow-up phase (CATSS-15) in order to capture a critical period of life where personality undergoes huge developmental processes related to adolescents' ill- and well-being. However, two different versions of the TCI, 125-item and 238-item versions, were used in the CATSS-15 in an epidemiological and a clinical assessment, respectively. The short version of the TCI was extracted from the long version in order to use data of all twins who completed the TCI. Thus, a first step in this study is to test the convergent reliability of these two versions and the validity of the extracting procedure.

The aims of the present study were:

1. to provide distributions (means and standard deviations) and to investigate the convergent reliability (*Cronbach's alpha*) of the long and short versions of the TCI in the CATSS and the validity of the extracting procedure (correlation).
2. to compare the TCI dimensions of the CATSS-twins to previously published data of adolescents from the Swedish general population.
3. to investigate the genetic structure of Cloninger's model in adolescence by twin modeling.

## Methods

### Ethic statement

Subjects are protected by informed consent process – they are informed of what is being collected and repeatedly given the option to withdraw their consent and discontinue their participation. All adolescents in the study had written consent from parents, caretakers, or guardians to participate in the study. The study has ethical approval from the Karolinska Institute Ethical Review Board: CATSS-15 Dnr: 2009/1599-32/5, DOGSS Dnr: 03-672 and 2010/1356/31/1.

### Sample and procedure

#### The CATSS (Baseline)

Since 2004, parents of all twins in Sweden have been interviewed via telephone by lay interviewers who, after a brief introduction to child and adolescent psychiatry and twin research, used a computerized version of the Autism – Tics, AD/HD and other Comorbidities inventory (A-TAC) [38], [39]. The A-TAC is a comprehensive telephone interview for evaluating symptoms and problem loads of a broad range of possible overlapping neurodevelopmental and behavioral disorders. For the baseline assessment of the CATSS, the twins' parents were contacted in connection to the twins' 9th birthday or 12th birthday for the telephone interview referred to as CATSS 9/12.

#### The CATSS-15 (Follow up)

**Figure 1:**
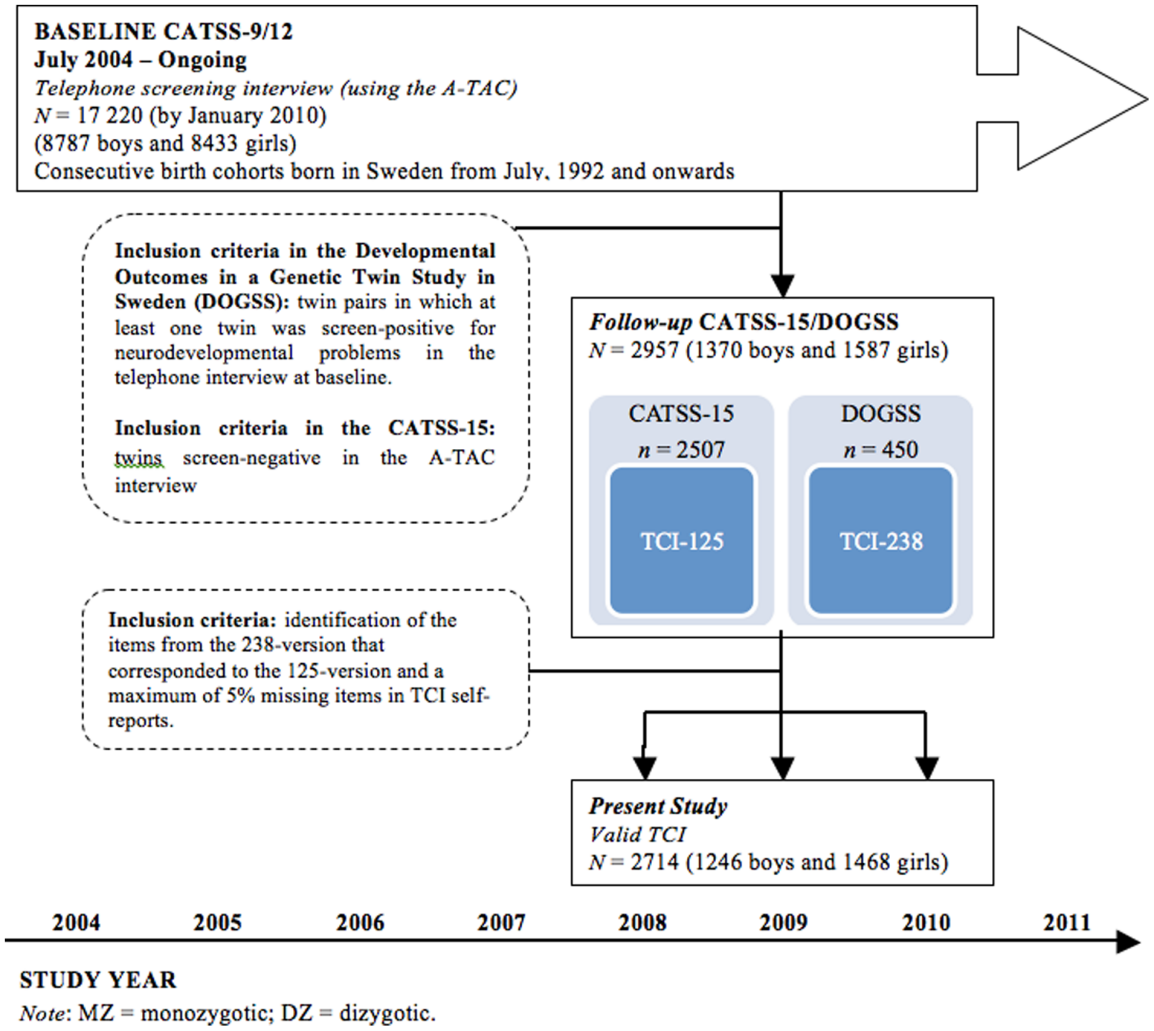
General study design of the Child and Adolescent Study in Sweden (CATSS).

The twins were contacted again when they were 15 years of age (CATSS-15) – the sample used in the present study. The CATSS-15 study consists of questionnaires that were sent home to the twins by mail (overall response rate 48%), including the short version (125 items) of the TCI. A thorough clinical follow-up study was also carried out of a selected part of the 15-year-old twins, creating a second sample that was included in the present study. In this clinical follow-up study, children who had been screened positive for different neurodevelopmental problems in the A-TAC interview at age 9/12, their co-twins, and matched controls were enrolled in a study called the Developmental Outcomes in a Genetic Twin Study in Sweden (DOGSS). The overall response rate in the DOGSS was over 60% with a tendency towards a higher response rate in controls and somewhat lower in the screened positive children. Participants in the DOGSS were asked to complete the longer version of the TCI (238 items) from which the shorter 125-items version could be extracted. This extracting procedure enabled the amalgamation of the CATSS-15- and the DOGSS-twins, which allowed us to achieve about the same response rate among children with psychosocial problems – a subgroup that is often under-sampled in population-based self-report studies – as what is found for normal children. Only twins from the CATSS-15 and the DOGSS who had no more than 5% missed TCI-items were included in the final sample. This procedure left a total of 2,714 twins (369 DOGSS-twins and 2,345 CATSS-15-twins) for individual comparisons (878 monozygotic, 885 same-sex dizygotic, 638 different-sex dizygotic, and 313 of unknown zygosity) and 423 monozygotic pairs and 408 same-sex dizygotic pairs for twin comparisons. See Figure 1.

#### The Swedish general population groups

We used data collected in two different published studies, Brändström [3] and Garcia [29], to compare the TCI data obtained in CATSS-15 and DOGSS with Swedish adolescents from the general population. In both studies on the general population the longer TCI was used; thus, responses for the TCI-125 were extracted from the long version by the same procedure as for the DOGSS data. The samples consisted of 332 adolescents (161 boys and 171 girls, between the ages of 15 and 16) from the Brändström study and 399 (198 boys and 201 girls, between the ages of 15 and 18) from the Garcia study.

### Measures

#### The Temperament and Character Inventory (TCI) [1]

Both versions of the TCI used in the present study measure the seven dimensions of the psychobiological model of personality. The dimensions are computed by summarizing binary answers (*true*  = 1, *false*  = 0) to a series of statements (some of which are reversely worded) for each dimension. Examples of questions from the four temperament dimensions are: Harm Avoidance, “I often feel tense and worried in unfamiliar situations, even when others feel there is little to worry about”; Novelty Seeking, “I often try new things just for fun or thrills, even if most people think it is a waste of time”; Reward Dependence, “I like to discuss my experiences and feelings openly with friends instead of keeping them to myself”; and Persistence, “I often push myself to the point of exhaustion or try to do more than I really can”. The three character dimensions: Self-directedness, “In most situations my natural responses are based on good habits that I have developed”; Cooperativeness, “I often consider another person's feelings as much as my own”; and Self-transcendence, “I sometimes feel so connected to nature that everything seems to be part of one living organism”.

#### Zygosity

Zygosity was determined by a validated algorithm based on five questions on twin similarity, derived from 571 pairs of twins with known zygosity. Only twins with more than a 95% probability of being correctly classified, compared to DNA-testing, were assigned zygosity by this method [40].

### Statistical methods

Using histograms, all TCI data (CATSS-15, DOGSS, Brändström, and Garcia) were considered to be normally distributed, thus all statistical tests regarding the first two aims were conducted using parametric methods in SPSS version 19. Regarding the first aim: *Cronbach's Alphas* were computed in order to test if the items of each TCI dimension yielded interpretable statements about individual differences [41], *means* and *standard deviations* were computed in order to provide useful comparison measures for the present and future studies, and *Pearson's correlation coefficients* were obtained for comparisons within and between the TCI dimension in all samples and for the two TCI versions in order to assess the accuracy of the extraction procedure. Regarding the second aim, means for all TCI dimensions (generated by both versions) were compared using *independent sample t-tests* for investigating differences between adolescents from the twin and general population. No raw data, only means and standard deviations were available from the Brändström study, thus comparisons between twins and this specific group, were conducted by one-sample *t*-tests.

The third aim was analyzed using twin methodology, which is basically a comparison of monozygotic-twins, who are genetically identical, and dizygotic-twins who, on average, share 50% of their segregating alleles. As a first step, intraclass correlation (ICC) coefficients for the seven dimensions in the TCI were calculated separately for monozygotic-twins and same-sex dizygotic-twins. As a second step, we performed univariate genetic analyses, using a model-fitting approach with structural equation-modeling techniques, using Mx [42] and SAS 9.3 software. By comparing the difference of ICC coefficients between monozygotic and dizygotic-twins it is possible to disentangle the genetic and environmental contribution to a trait. The genetic and environmental contributions are partitioned into three variance components: genetic factors (A), shared environmental factors that make the twins similar (C) and non-shared, unique environmental factors that make the twins dissimilar (E).

## Results

### Reliability, means, standard deviations and correlation coefficients

The reliability coefficients (*Cronbach's α*) for the TCI dimensions across all four samples and TCI versions were acceptable, with the exception of the temperament dimension of Reward Dependence and Persistence derived from the 125-item version. This was, however, similar to the 238-item version, in which Reward Dependence and Persistence also showed the lowest alphas. Moreover, the average *Cronbach's α* for the seven dimensions of the TCI 238-item version in the DOGSS was .77, while the average *Cronbach's α* for the extracted 125-item version in this sample was .71. The drop in reliability, average *Cronbach's α*, was even smaller for the Brändström (from.74 in the long version to .70 in the short version) and the Garcia studies (from .75 in the long version to .70 to the short version). Hence, the use of the common items in order to compute the short version's TCI dimensions seem to be nearly as reliable as using all items of the long version. See Table 1 for *Cronbach's α* and Table 2 for means and standard deviations for all TCI dimensions among samples (the statistically significant differences between samples indicated in Table 2 will be addressed in the comparison between twins and general population groups section). Overall the drop in reliability of the short version was small and inconsequential for the seven dimensions of the TCI.

**Table 1:** *Cronbach*'*s α* for the seven TCI dimensions in the CATSS-15, DOGSS, Brändström [3] and Garcia [29] study groups.

	*n*	Novelty Seeking	Harm Avoidance	Reward Dependence	Persistence	Self-directedness	Cooperativeness	Self-transcendence	*Mean Cronbach's α*
TCI-238									
DOGSS	369	.77	.84	.68	.61	.82	.83	.83	.77
Brändström	332	.73	.82	.64	.60	.80	.84	.79	.74
Garcia	399	.72	.85	.66	.62	.81	.82	.82	.75
TCI-125									
CATSS-15	2345	.66	.81	.46	.66	.81	.72	.78	.70
DOGSS^▾^	369	.70	.81	.57	.59	.79	.76	.79	.71
CATSS-15 & DOGSS^▾^	2714	.67	.81	.48	.66	.80	.72	.78	.70
Brändström^▾^	332	.65	.81	.52	.60	.77	.79	.76	.70
Garcia^▾^	399	.61	.82	.55	.61	.80	.75	.79	.70

*Note*: ^▾^Those 125 items identified from the 238-item TCI versions.

**Table 2:** Means and standard deviations in the seven TCI dimensions in the CATSS-15, DOGSS, Brändström [3] and Garcia [29] study groups.

	*N*	Novelty Seeking	Harm Avoidance	Reward Dependence	Persistence	Self-directedness	Cooperativeness	Self-transcendence
TCI-238 version								
DOGSS	369	22.50±5.97	**15.81±6.41^♀, B^**	**13.65±3.89^♀^**	4.04±2.00	**27.62±6.89^♂, B, G^**	**29.13±6.45^♀, B, G^**	**11.63±5.68^♀^**
Brändström	332	22.84±5.62	14.88±6.53	13.75±3.73	3.84±1.90	24.85±6.78	27.38±7.00	**15.52±5.92^D^**
Garcia	399	**22.36±5.52^♀^**	**15.23±6.78^♀^**	**14.33±3.81^♀,^^D^**	4.05±2.08	24.52±7.09	**27.55±6.73^♀^**	**13.45±5.91^D^**
TCI-125 version								
CATSS-15	2345	**10.88±3.45^♀^**	**8.69±4.41^♀^**	**8.68±2.36^♀, B, D^**	**2.56±1.62^B^**	**17.57±4.60^♂, B, D G^**	**18.45±3.71^♀, B, D, G^**	**3.44±2.92^♀^**
DOGSS^▾^	369	10.85±3.69	**9.73±4.50^♀, B, C, G^**	**8.22±2.64^♀^**	**2.44±1.52^B^**	**16.40±4.69^♂,^^B, G^**	**17.85±4.09^♀,^^B, G^**	**3.85±3.08^♀,^^C^**
CATSS-15 & DOGSS^▾^	2714	10.88±3.48	8.83±4.43	8.62±2.41	2.54±1.60	17.41±4.63	18.37±3.77	3.50±2.94
Brändström^▾^	332	**11.73±3.47^C, D, T^**	**8.91±4.60^C^**	8.25±2.58	2.23±1.54	14.60±4.72	16.36±4.65	**6.11±3.44 ^D, C, T^**
Garcia^▾^	399	**11.45±3.31^C, T^**	**8.87±4.65^♀^**	**8.60±2.65^♀^**	2.43±1.55	14.26±5.09	16.79±4.38^♀^	**5.15±3.47 ^C, D, T^**

*Note*: ^▾^Common items from the 125-item and 238-item TCI versions; ^♀^ higher than boys in the same sample (*P*<.05); ^♂^ higher than girls in the same sample (*P*<.05); ^B^ higher than adolescents in Brändström study group (*P*<.05); ^C^ higher than adolescents in the CATSS-15 (*P*<.05); ^D^ higher than adolescents in the DOGSS (*P*<.05); ^G^ higher than adolescents in the Garcia study group (*P*<.05); ^T^ higher than the whole twin group (CATSS-15 & DOGSS) (*P*<.05).

Strong correlations (ranging between 0.90 and 0.96) between the dimensions of the long and the extracted short version of the TCI were found (Table 3). Moreover, similar correlation coefficients for the intercorrelation between the seven TCI dimensions were found for both versions (Table 3). Similar results were obtained for the Brändström (0.89–0.96) and the Garcia (0.88–0.96) study groups between the dimensions of the long and the extracted short version of the TCI (see Table S1 and S2 in File S1). These findings support the validity of the dimensions yielded by the extraction procedure.

**Table 3:** Correlations between TCI dimensions in the DOGSS. Correlation coefficients between the 125- and 238-item TCI dimensions showed in the black fields, those within the TCI-238 dimensions in white, and correlations within the TCI-125 dimensions in gray.

	NS	HA	RD	PS	SD	CO	ST
Novelty Seeking (NS)		−.18^***^	.11^*^	−.19^***^	−.16^**^	−.08	.12^*^
Harm Avoidance (HA)	−.17^**^		.05	−.09	−.41^***^	−.01	−.12^*^
Reward Dependence (RD)	.00	.11^*^		−.01	.09	.39^***^	.06
Persistence (PS)	−.36^***^	−.18^***^	.12^*^		.09	.06	.21^***^
Self-directedness (SD)	−.31^***^	−.42^***^	.14^**^	.30^***^		.33^***^	−.13^*^
Cooperativeness (CO)	−.21^***^	−.10^*^	.52^***^	.27^***^	.46^***^		−.03
Self-transcend ence (SD)	.02	.04	.19^***^	.14^**^	−.17^***^	.18^***^	

*Note*: * *P*<.05; ** *P*<.01;*** *P*<.001; *n* = 369.

### Comparisons between twins and general population groups

Twins in the DOGSS showed higher levels of Harm Avoidance (*t* (2724)  = −3.87, *P*<.001, 95% CI = −1.44, −0.47) and lower levels of Self-directedness (*t* (2724)  = 3.65, *P*<.001, 95% CI = 4.38, 1.46) and Cooperativeness (*t* (2724)  = 2.12, *P*<001, 95% CI = .03, 0.88) when compared to the non-clinical population of CATSS-15 twins. The whole twin sample (the amalgamation of CATSS-15 and DOGSS) was analyzed next (*t*-tests and twin analysis). See Table S3 in File S1 for the intercorrelations of the whole twin sample. This merged twin sample had higher scores in the character scales of Self-directedness and Cooperativeness compared to both the Brändström (Self-directedness: *t* (3112)  = 27.94, *P*<.001, 95% CI = 2.24, 2.58; Cooperativeness: *t* (3112)  = 25.94, *P*<.001, 95% CI = 1.67, 1.94) and the Garcia studies (Self-directedness: *t* (3111)  = 12.54, *P*<.001, 95% CI = 2.66, 3.65; Cooperativeness: *t* (3111)  = 7.62, *P*<.001, 95% CI = 1.17, 1.98). In contrast, twins had lower Self-transcendence than adolescents in both Brändström (*t* (3112)  = −47.73, *P*<.001, 95% CI = −2.51, −2.29) and Garcia study groups (*t* (3111)  = −10.25, *P*<.001, 95% CI = −1.97, −1.34). (See Table 2 for means and standard deviations of the merged group). It is important to point out that this analysis used multiple comparisons, which might lead to incorrectly rejecting the null hypothesis (i.e., Type I error) [43]. Nevertheless, the maximum number of tests being performed for the different version of the TCI was 10. Hence, a Bonferroni correction to the alpha level suggested that a level of .005 was required for findings being significant. As detailed above, all the findings regarding the comparisons between the CATSS-twins and adolescents from the general population had a *P*-value <.001. In sum, twins had scores about half a standard deviation higher in the Self-directedness and Cooperativeness character dimensions, while adolescents from the general Swedish population had higher scores in the Self-transcendence dimensions.

### The genetic structure of the seven TCI dimensions in the whole twin sample

The TCI dimension showing the lowest heritability was the character dimension of Self-directedness (.29), while the highest heritability was found for two of the temperament dimensions: Harm Avoidance (.50) and Persistence (.52). More importantly, the results showed a shared environmental influence for all of the character dimensions (Self-directedness = .22; Cooperativeness = .21; Self-transcendence = .11) and for one of the temperament dimensions (i.e., Reward Dependence = .11). See Table 4 for the details.

**Table 4:** Intraclass correlations (ICC) according to zygosity and estimates of genetic and environmental effects for the seven TCI [95% CI].

Dimension	Trait	MZ	DZ	A	C	E
		(*n* = 423 pairs)	(*n* = 408 pairs)	Additive Genetics	Common Environment	Unique Environment
TEMPERAMENT	Novelty Seeking	0.45	0.14	.44	.00	.56
		[.36–.52]	[.05–.24]	[.34–.51]	[.00–.07]	[.49–.64]
	Harm Avoidance	0.51	0.19	.50	.00	.50
		[.43–.57]	[.09–.28]	[.38–.56]	[.00–.09]	[.44–.57]
	Reward Dependence	0.45	0.30	.36	.11	.53
		[.38–.53]	[.21–.39]	[.14–.53]	[.00–.29]	[.46–.61]
	Persistence	0.56	0.11	.52	.00	.48
		[.49–.62]	[.01–.21]	[.45–.59]	[.00–.05]	[.41–.54]
CHARACTER	Self-directedness	0.52	0.36	.29	.22	.49
		[.44–.58]	[.27–.44]	[.09–.50]	[.04–.38]	[.43–.56]
	Cooperativeness	0.59	0.40	.38	.21	.41
		[.52–.65]	[.32–.48]	[.19–.57]	[.04–.37]	[.36–.47]
	Self-transcendence	0.51	0.31	.40	.11	.49
		[.43–.58]	[.22–.39]	[.19–.56]	[.00–.28]	[.43–.56]

Note: MZ = Monozygotic; DZ = Dizygotic (same-sex).

## Discussion

In the present study, testing the reliability of both TCI versions included in the CATSS-15 and DOGSS follow up, as well as validation of the extraction procedure was of special interest. The first important finding of the present study is that the extraction procedure generated dimensions that were as reliable as those obtained by the longer version, both from the DOGSS and from the Brändström and Garcia studies as well. Our results also show that the TCI dimensions obtained by this procedure were highly correlated to those obtained with the longer version, suggesting that both the original version as well as the ”extracted short version” are equally reliable and could function as measures of personality dimensions in adolescents.

The second key-question of the present study was how similar/dissimilar Swedish twin adolescents are, compared to Swedish adolescents from the general Swedish population. Twins had scores about half a standard deviation higher in the Self-directedness and Cooperativeness character dimensions, while adolescents from the general Swedish population had higher scores in the Self-transcendence dimensions. Longitudinal studies in Finland have found that people increase in Self-directedness and Coooperativeness while decreasing in Self-transcendence as they mature from ages 20 to 45 years of age [23]. Hence the pattern we observed suggests that the twins are precocious in the maturation of their character organization when compared to singletons. This was the case for comparisons using both the long and the short versions. In other words, this difference was not dependent on the common item extraction procedure.

The twins also reported higher scores, compared to the Brändström study group, in the temperament dimensions of Reward Dependence and Persistence. It is known that among children, Reward Dependence, Persistence, Cooperativeness, and Self-directedness are positively related to intelligence and academic achievement [44], [45], which would suggest that twins might have better prerequisites for performing well in school and in academic life. However, other studies have found that twins have substantially lower IQs [46] and perform less well academically later in life [47] than singletons. These results can be explained, at least partly, with reduced prenatal growth and low birth weight of twins [47]. Nevertheless, studies using recent Danish cohorts of twins have found that twins show similar academic performance in adolescence when compared to singletons [48]. In a Swedish cohort, researchers also found that, despite male twins having slightly lower IQs compared to male singletons; twins had slightly better mean grade point averages in ninth grade, and had more often completed a university education in young adulthood [49]. The present study, in addition to the two studies conducted in Scandinavia, shows more positive outcomes among twins compared to singletons. It is plausible to suggest that the key factors that vary over time and between societies, such as quality of neonatal care and educational and social policies, might cause the different results from studies in other cultures [47].

The third and final aim was to investigate the contributions of genes and environment on the seven dimensions of Cloninger's psychobiological model of personality using same-sex twins from the CATSS-15 and DOGSS. The results showed that at least among adolescents there is a shared environmental influence on all of the character dimensions, but only on one of the temperament dimensions (i.e., Reward Dependence). Previous research about the etiology of personality dimensions in young adults [19] suggests a small shared environmental influence for Self-directedness and Cooperativeness, as well as for the temperament dimension of Reward Dependence. Moreover, studies among adults [20] have not found shared environmental influences behind the character dimensions. In contrast, the heritability behind the character dimensions in both study populations (i.e., [19], [20]) seems comparable and are all within the confidence intervals shown in the present study. Our findings regarding the existence of shared environmental factors behind the character dimensions in adolescence, together with small shared environmental effects on character among young adults and no shared environmental effects on character among adults suggest that there is a shift in type of environmental influence from adolescence to adulthood regarding character – adolescence appears to be a crucial developmental period in which to identify sociocultural influences on personality development and interventions may need to be tailored to each age group. Dosenbach, Petersen, and Schlaggar [50], for instance, show that prefrontal connections that underlay the ability to flexibly regulate impulses and decisions emerge by adolescence and continue to grow stronger into adulthood (see also [51]). Moreover, hormonal influences on the brain during puberty enhance sensitivity toward social stimuli [52].

### Limitations

In the present study we had no information regarding the adolescents' birth weights, or parents' ages, educations, or socioeconomic statuses. Although this information is accessible for the CATSS data, it was not obtainable for the general population groups, making this analysis impossible. These variables might be important to examine before we can actually suggest that twins might have advantages, with regard to character maturity, compared to the general population. Moreover, the sample used here was composed of data from the DOGSS, in which adolescents were selected by one or both of the twins being screen positive for neurodevelopmental problems. This suggests that the results presented here should be interpreted with caution. Nevertheless, we suggest that the inclusion of the DOGSS-twins allowed a more robust comparison because this is a subgroup that is often under-sampled in population-based self-report studies.

It is possible that our findings regarding the genetic structure of Cloninger's model of personality differ from those of earlier research because of differences in measurement. Most research has been done using the longer version of the TCI. Nevertheless, similar results to those obtained with the long version have been found using shorter versions (e.g., see [20], who used a 35-item version for measuring the character dimensions). In contrast, our results show that the extraction-item-procedure generated dimensions that were highly correlated, suggesting that they will produce similar results when the genetic structure of the model is investigated.

### Clinical implications

Research on the development of twins has been described as a “natural experiment” and is commonly used to test “hypotheses about health, development, and behavior in general” ([49], p. 591). Our findings, that twins according to the TCI seem to have a more mature character than adolescents from the general population, might explain why twins, despite prenatal adversity, do not display more adult morbidity and mortality than singletons [53]. If so, twin studies might need to include measures of character when testing hypotheses related to health and other human behavior.

Together with recent prospective studies showing increases in Self-directedness and Cooperativeness (which is an indicator of increasing responsibility and relatedness) with age (from 20 to 45 [20]), our results regarding the genetic structure suggest that character and personality are not fixed. Low character maturity at a young age is a good indicator that the individual is at a substantial risk of developing a harmful lifestyle for both himself and others [2]. If the common environment influences Self-directedness and Cooperativeness, it might be crucial to identify individuals with low scores in Self-directedness and Cooperativeness at an early age in order to introduce interventions that might help to increase their sense of responsibility and relatedness. Magen [54] suggests that interventions that influence adolescents to give and receive help (e.g., peer counseling) strengthens self-acceptance and fosters adolescents' capacity to experience moments of happiness and identity formation. The benefits from such interventions are greater “self-esteem, sense of purpose and worth, feelings of accomplishment and mastery, and satisfying interaction with other human beings” ([54], p. 192). These benefits are, at least in part, good definitions of Self-directedness and Cooperativeness.

### Suggestions for future research

The character and temperament dimensions are higher order dimensions composed of lower order facets. There are both advantages and disadvantages when personality is investigated in terms of broad dimensions. One advantage is that each TCI dimension represents wide-ranging descriptions of personality, allowing the prediction of many outcomes (e.g., personality disorders). On the other hand, the aggregation of the lower order facets in one higher order dimension results in a loss of information – information that might be useful for psychological description, prediction, and explanation [55]. The Congruent Second Nature vs. Bad Habits Self-directedness facet of the TCI, for instance, can actually measure self-discipline. Among adolescents, self-discipline outdoes IQ when predicting academic performance [56], [45]. In light of the studies showing lower IQ among twins compared to singletons [47], studies showing equal or even slightly better academic performance among twins compared to singletons [48], [49], and the results presented here showing higher Self-directedness scores among twins compared to general population groups, it is reasonable to suggest tentatively that twins might overcome having lower IQs as long as they develop a spectrum of goal-congruent, good habits so that they automatically act in accordance with their long-term values and goals, as high scorers in the Congruent Second Nature vs. Bad Habits Self-directedness facet are defined. Thus, studies investigating the genetic structure of the lower order facets are needed to suggest which facets might need and/or can be targeted by interventions.

## Conclusions

The extraction of common items in the long and short TCI versions did not alter the psychometric properties of the seven personality dimensions, which were strongly correlated to their corresponding dimensions derived by the long TCI version, hence suggesting high reliability and correlation for the short version dimensions created by this procedure. Compared to adolescents from the general population, twins may have advantages pertaining character maturity. A more mature character might explain why twins, despite adversities related to prenatal and birth problems, sometimes show equal health outcomes or sometimes slightly better cognitive performance than singletons. Even if the confidence intervals are large and partly overlapping, our study supported the theoretical notion behind Cloninger's model and studies of longitudinal development showing that temperament traits are under strong genetic influence and are different from character scales that describe the development of regulatory cognitive-emotional strategies with a more complex etiology, including significant common environmental effects.

## Supporting Information

File S1 **Table S1-Table** **S3.** Table S1. Correlations between TCI dimension in the Brändström study. Correlation coefficients between the 125- and 238-items TCI dimensions showed in the black fields, those within the TCI −238 dimensions in white, and correlations within the TCI-125 dimensions in grey. Table S2. Correlations between TCI dimension in the Garcia study. Correlation coefficients between the 125- and 238-items TCI dimensions showed in the black fields, those within the TCI −238 dimensions in white, and correlations within the TCI-125 dimensions in grey. Table S3. Correlations between TCI dimensions (short version) in the whole twin sample (CATSS-15 and DOGSS).
(DOCX)
