# Supplementary material for: Temperament and Character in the Child and Adolescent Twin Study in Sweden (CATSS): Comparison to the General Population, and Genetic Structure Analysis
Source: PLoS One. 2013 Aug 5;8(8):e70475. doi: 10.1371/journal.pone.0070475 (PMC3734246; doi:10.1371/journal.pone.0070475)
Supplement: File S1 — Table S1-Table S3. Table S1. Correlations between TCI dimension in the Brändström study. Correlation coefficients between the 125- and 238-items TCI dimensions showed in the black fields, those within the TCI −238 dimensions in white, and correlations within the TCI-125 dimensions in grey. Table S2. Correlations between TCI dimension in the Garcia study. Correlation coefficients between the 125- and 238-items TCI dimensions showed in the black fields, those within the TCI −238 dimensions in white, and correlations within the TCI-125 dimensions in grey. Table S3. Correlations between TCI dimensions (short version) in the whole twin sample (CATSS-15 and DOGSS). (DOCX) [file pone.0070475.s001.docx]

SUPPORTING INFORMATION

Temperament and Character in the Child and Adolescent Twin Study in Sweden (CATSS): Validation, Comparison to the General Population, and Genetic Structure Analysis

**Table S1.**

|  | NS | HA | RD | PS | SD | CO | ST |
| --- | --- | --- | --- | --- | --- | --- | --- |
| Novelty Seeking (NS) | **.90^***^** | -.14^**^ | -.04 | -.28^***^ | -.32^***^ | -.20^***^ | .07 |
| Harm Avoidance (HA) | -.19^***^ | **.95^***^** | .15^**^ | -.21^***^ | -.35^***^ | -.06 | .03 |
| Reward Dependence (RD) | .15^**^ | .03 | **.93^***^** | .05 | .05 | .41^***^ | .04 |
| Persistence (PS) | -.25^***^ | -.18^***^ | .01 | **.89^***^** | .23^***^ | .20^***^ | .18^***^ |
| Self-directedness (SD) | -.13^*^ | -.47^***^ | .07 | .20^***^ | **.93^***^** | .36^***^ | -.28^***^ |
| Cooperativeness (CO) | -.04 | -.05 | .49^***^ | 12^*^ | .39^***^ | **.96^***^** | .10 |
| Self-transcendence (SD) | .13^*^ | -.13^*^ | .18^***^ | .15^**^ | -.08 | .07 | **.91^***^** |

*Note*: ; * *P*< .05; ** *P*< .01,*** *P*< .001; *N* = 332.

**Table S2.**

|  | NS | HA | RD | PS | SD | CO | ST |
| --- | --- | --- | --- | --- | --- | --- | --- |
| Novelty Seeking (NS) | **.88^***^** | -.19^***^ | .19^***^ | -.16^**^ | .04 | .05 | .08 |
| Harm Avoidance (HA) | -.18^***^ | **.95^***^** | .18^**^ | -.18^***^ | -.43^***^ | -.06 | -.02 |
| Reward Dependence (RD) | .20^***^ | .16^**^ | **.92^***^** | -.04 | .06 | .45^***^ | .08 |
| Persistence (PS) | -.18^***^ | -.14^**^ | .08 | **.92^***^** | .15^**^ | .06 | .20^***^ |
| Self-directedness (SD) | .05 | -.48^***^ | .13^*^ | .26^***^ | **.93^***^** | .37^***^ | -.18^***^ |
| Cooperativeness (CO) | .12^*^ | -.07 | .56^***^ | .16^***^ | .41^***^ | **.96^***^** | .03 |
| Self-transcendence (SD) | .04 | .05 | .22^***^ | .14^**^ | -.12^*^ | .15^**^ | **.91^***^** |

*Note*: * *P* < .05; ** *P* < .01,*** *P* < .001; *N* = 399.

**Table S3.**

|  | NS | HA | RD | PS | SD | CO | ST |
| --- | --- | --- | --- | --- | --- | --- | --- |
| Novelty Seeking (NS) | - |  |  |  |  |  |  |
| Harm Avoidance (HA) | -.24^***^ | - |  |  |  |  |  |
| Reward Dependence (RD) | -.01 | .07^***^ | - |  |  |  |  |
| Persistence (PS) | -.20^***^ | -.18^***^ | .12^***^ | - |  |  |  |
| Self-directedness (SD) | -.21^***^ | -.41^***^ | .11^***^ | .19^***^ | - |  |  |
| Cooperativeness (CO) | -.18^***^ | -.06^**^ | .40^***^ | .20^***^ | .38^***^ | - |  |
| Self-transcendence (SD) | .13^***^ | .00 | .05^*^ | .19^***^ | -.32^***^ | .02 | - |

*Note*: * *P*<.05; ** *P*<.01;*** *P*<.001; *N* = 2714.
